# Serum Cystatin C as a Marker of Renal Function in Critically Ill Patients With Normal Serum Creatinine

**DOI:** 10.5812/numonthly.15224

**Published:** 2014-03-01

**Authors:** Mohammad Mahdi Sagheb, Soha Namazi, Bita Geramizadeh, Amin Karimzadeh, Mohammad Bagher Oghazian, Iman Karimzadeh

**Affiliations:** 1 Nephrology Urology Research Center and Department of Internal Medicine, Shiraz University of Medical Sciences, Shiraz, IR Iran; 2 Department of Clinical Pharmacy, Faculty of Pharmacy, Shiraz University of Medical Sciences, Shiraz, IR Iran; 3 Transplant Research Center and Department of Pathology, Shiraz University of Medical Sciences, Shiraz, IR Iran; 4 Department of Dermatology, Imam Khomeini Hospital, Jundishapur University of Medical Sciences, Ahvaz, IR Iran; 5 Department of Clinical Pharmacy, Faculty of Pharmacy, Tehran University of Medical Sciences, Tehran, IR Iran

**Keywords:** Critically Ill, Patients, Creatinine, Cystatin C, Acute Kidney Injury

## Abstract

**Background:**

Serum creatinine as a classic marker of renal function has several limitations in the detection of renal dysfunction.

**Objectives:**

This study assessed the validity of serum cystatin C as a marker of renal function in critically ill patients with normal serum creatinine.

**Patients and Methods:**

Eighty adult patients referred to intensive care units with serum creatinine levels < 1.5 mg/dL and without hemodynamic instability were chosen and their serum creatinine and cystatin C levels were measured. A 24-hour urine sample was collected to calculate creatinine clearance (Ccr). Renal dysfunction was defined as Ccr < 80 mL/min/1.73 m^2^.

**Results:**

There were significant correlations between measured Ccr and 1/serum creatinine (R = 0.51, P < 0.001) and 1/serum cystatin C (R = 0.25, P = 0.028). The difference between false negative rates of serum creatinine (93.33%) and cystatin C (80%) in the detection of renal dysfunction was significant (P = 0.032). Receiver operating characteristic curve analysis illustrated that area under the curve of serum creatinine and cystatin C for detecting renal dysfunction were 0.711 and 0.607, respectively; however, this difference was not significant (P = 0.222).

**Conclusions:**

Our data demonstrated that serum cystatin C is not superior to serum creatinine in the early detection of renal dysfunction in critically ill patients.

## 1. Background

Acute kidney injury (AKI) comprises several syndromes that are associated with a sudden and persistent, but potentially reversible decrease in renal function. AKI occurs in up to 30% of all patients admitted to the intensive care units (ICU). Despite many advances in medicine during the past two decades, the mortality and morbidity of AKI in the ICU have remained high without significant improvement (1). This condition associates with mortality rates of 50% to 90% in ICU patients requiring renal replacement therapy (2). In clinical practice, the detection of AKI is based on an increase in serum creatinine (3). However, there are several drawbacks to the use of creatinine. Many non-renal factors might influence creatinine level, such as age, gender, race, protein intake, muscle mass, infections, and inflammatory status. Furthermore, serum creatinine does not accurately reflect the glomerular filtration rate (GFR) during AKI (4). Underestimation of renal function may contribute to inappropriate dosing and increased risk of adverse reactions of medications like many antibiotics that require dose adjustment in renal dysfunction (5). Cystatin C, a non-glycosylated protein with cysteine proteinase inhibitor activity, has been considered as a new marker of renal function. Because of its constant rate of production by all nucleated cells, the cystatin C serum level is only determined by GFR. Moreover, its concentration is not influenced much by age, gender, muscle mass, infections, and inflammatory or liver diseases (6, 7). Several studies demonstrated the superiority of serum cystatin C in comparison with creatinine in the detection of minor GFR reduction (8-10). To the best of our knowledge, data regarding the validity of cystatin C as a marker of renal function in ICU patients are inconclusive and most studied populations were small too.

## 2. Objectives

The purpose of this study was to assess serum cystatin C as a marker of early AKI detection in critically ill patients with normal serum creatinine.

## 3. Patients and Methods

This cross-sectional study was conducted from early September 2006 to late September 2008 at three surgical, neurosurgical, and medical ICUs of Namazi Hospital, a multispecialty healthcare university setting in Shiraz, Iran. The Institutional Review Board (IRB) and the Medical Ethics Committee of Shiraz University of Medical Sciences approved the study and written informed consent was obtained from all patients or their families.

At first step, patients older than 18 years with an indwelling urinary catheter, serum creatinine levels < 1.5 mg/dL, and with a hospital and ICU stay < 1 week were included in the study. Then, patients who were hemodynamically unstable (mean arterial pressure < 70 mmHg, systolic blood pressure < 90 mmHg, or under vasoactive medication therapy) (11), recovering from AKI or developing AKI, required renal replacement therapy, or transferred from another ICU were excluded from this study. Due to potential impacts on the serum cystatin C level, patients with aortic aneurism (12), overt hypothyroidism or hyperthyroidism (13, 14), and patients receiving glucocorticoids (15) were also excluded. Finally, we excluded patients receiving cimetidine or trimethoprim to avoid their inhibitory effects on tubular secretion of creatinine (16). Furthermore, no patient received diuretics. Demographic and clinical characteristics of patients, including age, sex, weight, height, duration of hospitalization before ICU admission, length of ICU stay preceding recruitment into the study, admission diagnosis, and mechanical ventilation dependency were recorded. Acute physiology and chronic health evaluation (APACHE II) score (17) was calculated for each patient within the first 24 hours of ICU admission. A 24-hour urine sample was collected to calculate creatinine clearance (Ccr) (16). A 10 ml serum sample was drawn from each patient in the morning just after the end of urine collection to determine serum creatinine, urea, and albumin by a profile analyzer (Prestige 24I, Nippon, Tokyo, Japan). The normal reference value for serum creatinine ranges from 0.8 to 1.3 mg/dL for men and 0.6 to 1.2 mg/dL for women (Elitech, Sees, France). The enzyme-linked immunosorbent assay (ELISA) kit (Biovendor, Brno, Czech Republic) was used for the determination of serum cystatin C level. For serum cystatin C, the normal reference values are 0.8-1.1 mg/L for men and 0.6-1.1 mg/L for women. In addition to Ccr corrected for the body surface area (BSA), renal function was also assessed by three Cockcroft-Gault (CG) with ideal body weight (18), original (19), and simplified modification of diet in renal disease (MDRD) (20) formulas, which all were normalized for BSA. Renal dysfunction was defined as measured Ccr or calculated GFR < 80 mL/min/1.73 m^2^.

### 3.1. Statistical Analysis

Categorical data were expressed as percentage. Continuous variables were reported as mean ± standard deviation (SD). Pearson's correlation test was used to assess the possible correlation between studied markers of renal function (serum creatinine and cystatin C) as well as between inverses of creatinine and cystatin C and Ccr. Comparing false negative percentages of renal dysfunction obtained from creatinine and cystatin C values was performed by Chi-square test. Multivariate logistic regression analysis was used to compare demographic and clinical characteristics of patients with and without renal dysfunction. Odds ratios (OR) and their 95% confidence intervals (CI) were calculated for each variable. The diagnostic value of serum creatinine and cystatin C in identifying GFR < 80 mL/min/1.73 m^2^ was assessed by the receiver operating characteristic (ROC) curves of sensitivity and specificity and the data were expressed as area under the curve (AUC) and their 95% CI. P-values less than 0.05 were considered as statistically significant. All statistical analyses but ROC curve, were performed by the statistical package for the social sciences (SPSS) version 11.5 (SPSS Inc., Chicago, IL, USA). ROC curve analysis was executed by the MedCalc version 8.0. 

## 4. Results

**Figure 1.**
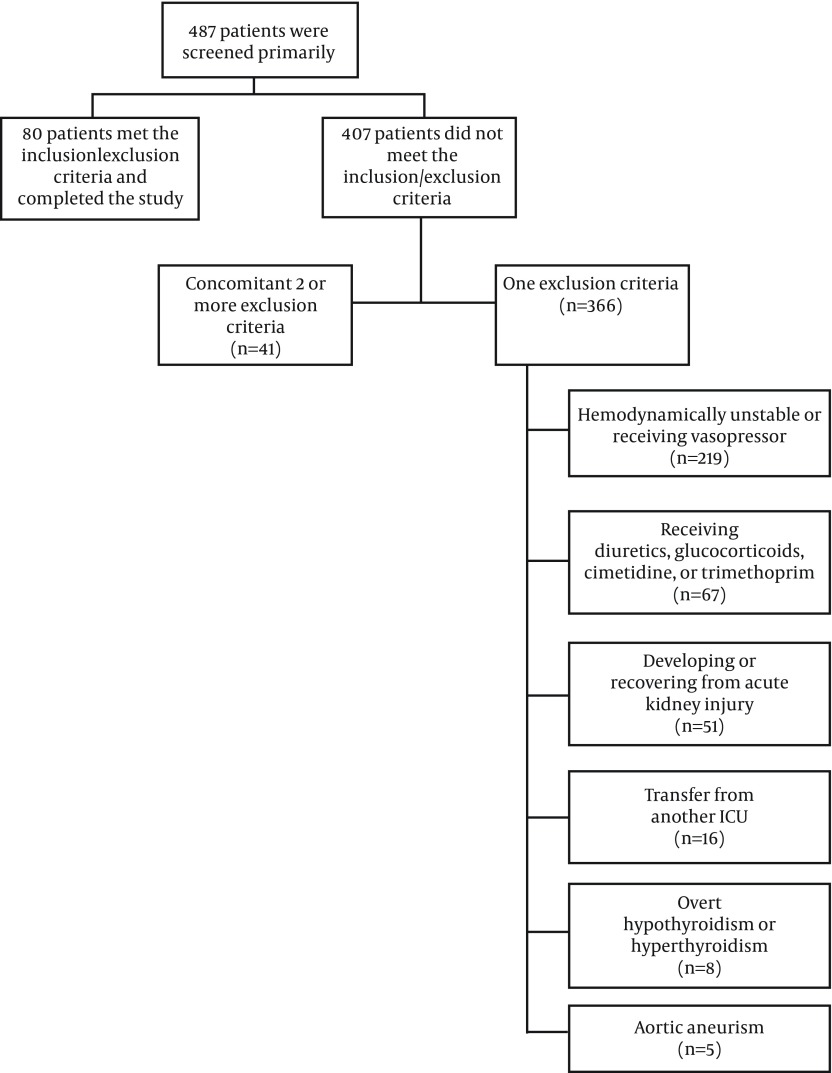
Flowchart of Patient Recruitment

During two years, 80 patients, including 55 males and 25 females with mean ± SD age of 43.53 ± 18.31 years (minimum-maximum, 18-77 years) who met the inclusion criteria were recruited into this study (Figure 1). Their mean ± SD APACHE II score was 8.14 ± 5.24 (minimum-maximum, 2-27). The mean ± SD duration of patients stay in ICU preceding recruitment into the study was 3.04 ± 1.76 days. Table 1 lists the admission diagnosis of the study population. Neurologic disease (18.75%) was the most common admission diagnosis followed by malignancy (15%) and multiple blunt trauma without head injury (11.25%). Forty two (52.5%) patients were mechanically ventilated. The mean ± SD serum creatinine, cystatin C, 24-hour urine volume, and measured Ccr of the study population were 0.89 ± 0.25 mg/dL, 0.88 ± 0.41 mg/L, 2983.07 ± 1612.48 mL, and 65.07 ± 36.19 mL/min/1.73 m^2^, respectively.

**Table 1.** Admission Diagnosis of the Patients Included in the Study (n = 80) ^a^

Diagnosis	No. (%)
**Neurologic disease**	15 (18.75)
**Malignancy**	12 (15)
**Multiple blunt trauma without head injury**	9 (11.25)
**Drug/alcohol poisoning**	7 (8.75)
**Cardiovascular disease**	6 (7.5)
**Multiple blunt trauma with head injury**	5 (6.25)
**Head injury**	5 (6.25)
**Abdominal surgery**	4 (5)
**Metabolic disease**	4 (5)
**Pulmonary disease**	4 (5)
**Infectious disease**	3 (3.75)
**Penetrating trauma without head injury**	2 (2.5)
**Electrical injury**	2 (2.5)
**Other**	2 (2.5)

^a^Including gastrointestinal bleeding (1 case) and compartment syndrome due to snakebite (1 case).

There were statistically significant correlations between measured Ccr and 1/serum creatinine (R = 0.51, P < 0.001) as well as 1/serum cystatin C (R = 0.25, P = 0.028). According to measured Ccr, 60 (75%) patients had renal dysfunction (Ccr < 80 mL/min/1.73 m^2^). Among these patients, serum creatinine and cystatin C were above normal level only in 4 (6.67%) and 12 (20%), respectively. In other words, the false negative rates of serum creatinine and cystatin C in the detection of renal dysfunction were 56/60 (93.33%) and 48/60 (80%), respectively. This difference was statistically significant (P = 0.032).

A comparison between different demographic and clinical characteristics of patients with and without renal dysfunction is provided in Table 2. No statistically significant difference among studied variables was identified. 

**Figure 2.**
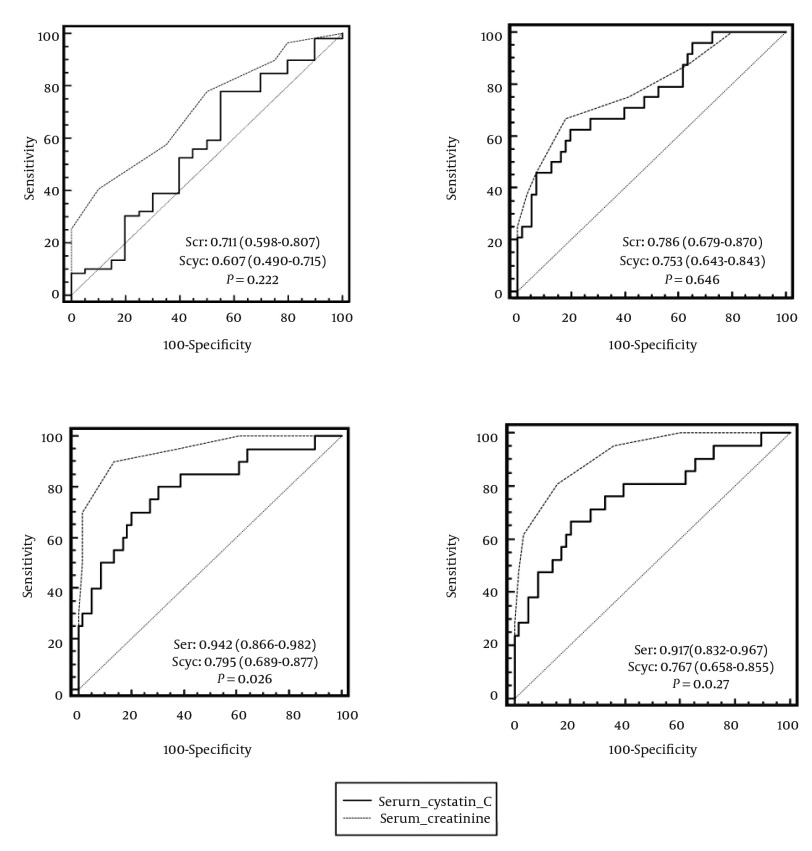
ROC Curves of Sensitivity and Specificity of Serum Creatinine and Cystatin C for Detection of GFR < 80 mL/min/1.73 m^2^ by Measured Creatinine Clearance (a), Cockcroft-Gault (b), Original (c), and Simplified (d) Modification of Diet in Renal Disease (MDRD) Equations. AUC (95% CI) and P value for Each Formula Are Provided within the Related Curves.

ROC curves of sensitivity and specificity of serum creatinine and cystatin C for detection GFR < 80 mL/min/1.73 m^2^ measured or calculated by different formulas are demonstrated in Figure 2. In the case of renal dysfunction (measured Ccr < 80 mL/min/1.73 m^2^), the AUC (95% CI) for serum creatinine was 0.711 (0.598-0.807) and for serum cystatin C was 0.607 (0.490-0.715). The difference between AUC of these two markers (0.104, 95% CI = 0.063-0.270) was not statistically significant (P = 0.222). To diagnose GFR < 80 mL/min/1.73 m^2^ calculated by the CG equation (with ideal body weight), the AUC of serum creatinine (0.786, 95% CI = 0.679-0.870) was greater than that of serum cystatin C (0.753, 95% CI = 0.643-0.843). However, this difference was not statistically significant (P = 0.646). In the case of original and simplified MDRD equations, there was a statistical significant difference between AUC of serum creatinine (0.942, 95% CI = 0.866-0.982 and 0.917, 95% CI = 0.832-0.967) and serum cystatin C (0.795, 95% CI = 0.689-0.877 and 0.767, 95% CI = 0.658-0.855) for detecting GFR < 80 mL/min/1.73 m^2^ (P = 0.026 and P = 0.027, respectively). In other words, the accuracy of serum creatinine for detection of GFR < 80 mL/min/1.73 m^2^ calculated by the original and simplified MDRD formulas was statistically higher than that of serum cystatin C.

**Table 2.** Different Demographic and Clinical Characteristics of Patients With and Without Renal Dysfunction (n = 80) ^a,^^b, c^

	Measured Ccr Below 80 mL/min/1.73 m^2^, (n = 59)	Measured Ccr Above 80 mL/min/1.73 m^2^, (n = 21)	OR (95% CI)	P value
**Sex, No. (%)**			0.874 (0.107-7.111)	0.9
Male	40 (67.79)	15 (71.43)		
Female	19 (32.2)	6 (28.57)		
**Age, y**	45.14 ± 18.66 [18-77]	38.8 ± 16.81 [21-73]	1.033 (0.983-1.086)	0.204
**Body surface area, m** ^**2**^	1.73 ± 0.17 [1.36-2.13]	1.76 ± 0.17 [1.49-2.08]	0.016 (0.001-25.788)	0.274
**Body mass index, kg/m** ^**2**^	23.52 ± 5.17 [13.6-36.8]	22.75 ± 3.43 [16-29.3]	1.118 (0.867-1.44)	0.39
**Duration of hospitalization before ICU admission, d**	1.69 ± 1.38 [1-6]	1.6 ± 1.27 [1-6]	1.28 (0.724-2.263)	0.397
**Length of ICU stay preceding recruitment into the study, d**	3.07 ± 1.85 [1-6]	2.95 ± 1.5 [1-7]	1.022 (0.7-1.492)	0.911
**APACHE II score**	8.61 ± 5.74 [2-27]	6.75 ± 3.08 [3-13]	1.154 (0.958-1.39)	0.133
**Mechanical ventilation dependency (%)**			0.401 (0.082-1.97)	0.26
Dependent	32 (54.24)	10 (47.62)		
Independent	27 (45.76)	11 (52.38)		

^a^ All data represented as Mean ± SD, Minimum-Maximum, Or Percentage.

^b^ Abbreviations: APACHE, acute physiology and chronic health evaluation; Ccr, creatinine clearance; CI, confidence interval; ICU, Intensive care unit.

^c^ mean ± SD [Minimum-Maximum].

## 5. Discussion

Early identification of renal injury can prevent progression to AKI and decrease subsequent morbidity, mortality, and additional costs (21). Failure of most current pharmacological interventions for AKI may be partially due to the delay in the diagnosis of renal injury (1). Individuals with initial normal serum creatinine may benefit more from early detection of renal dysfunction because they are generally overlooked compared with those with elevated creatinine levels at the baseline. Despite direct measurement of GFR by exogenous substances such as inulin or iothalamate -the gold standard method-, it is not practical in clinical settings (16). Identifying an endogenous marker of renal function with appropriate accuracy is an urgent demand. The results of a meta-analysis on 13 studies demonstrated that serum cystatin C appears to be a good biomarker for prediction of AKI development both overall and across a range of subgroups (22). In the current study, we examined the hypothesis that serum cystatin C is more accurate than serum creatinine for detection of early AKI, defined as GFR < 80 mL/min/1.73 m^2^, in critically ill patients. Our preliminary findings suggested that serum cystatin C has no superiority and advantage over serum creatinine in early detection of AKI in ICU patients.

The inverse correlation between serum creatinine (1/serum creatinine) and measured Ccr in our survey was statistically significant (P < 0.001) and the correlation coefficient (R = 0.51) was within the range (R = 0.5-0.89) reported from other studies (23-27). The mean correlation coefficient for the inverse of serum cystatin C (1/serum cystatin C) from 36 data sets (R = 0.816, 95% CI = 0.804-0.826) (28) was much higher than that from the current study (R = 0.25). Similarly, Khorgami et al. demonstrated the statistically significant correlation (P < 0.001) between serum cystatin C and simplified MDRD as well as cystatin C-based formula in chronic hemodialysis individuals with correlation coefficients of -0.46 and -0.87, respectively (29). This difference can be partially explained by the characteristics of patients. Studies assessed in the meta-analysis by Dharnidharka et al. (28) as well as Khorgami et al. study (29) were predominantly conducted in individuals with generally stable clinical condition while our study population were critically ill. Despite the fact that only hemodynamically stable subjects were selected, it seems impossible to detect and control occasional alterations in kidney perfusion and GFR that are associated with transient fluctuations in blood pressure or changes in the rate of fluid administration. 

Our study showed that 93.33% of patients with normal serum creatinine had Ccr < 80 mL/min/1.73 m^2^. The false negative rate of serum creatinine in the detection of renal dysfunction reported from similar studies in ICU patients varies from 42% up to 80% (30-33). The low sensitivity of creatinine in the detection of renal dysfunction in critically ill patients might be due to the lower creatinine production. Muscle loss due to the primary illness can be considered as the most plausible explanation for the depressed creatinine production. The other possible factors are inadequate dietary intake of creatine (the major source of creatinine) and impaired liver function, which is often present in ICU patients. In line with our results, Delanaye et al. (30) and Villa et al. (33) found that the false negative rate of serum cystatin C in the detection of renal dysfunction (Ccr < 80 mL/min/1.73 m^2^) in critically ill patients was significantly lower than that of serum creatinine.

The results of ROC curve analysis demonstrated that the accuracy of serum cystatin C in detection of renal dysfunction (Ccr < 80 mL/min/1.73 m^2^) was comparable to serum creatinine (AUC = 0.711 vs. 0.607, P = 0.222). The results of the studies comparing serum cystatin C and creatinine as markers of renal function in ICU patients are inconclusive. One study on 202 adult ICU patients in Finland reported that serum cystatin C rises as quickly as serum creatinine. In other words, serum cystatin C performed as well as serum creatinine in the detection of AKI in critically ill patients (34). Mazul-Sunko et al. found no statistically significant correlation between cystatin C plasma level (obtained on the day of ICU admission) and development of AKI in 29 critically ill patients with sepsis (35). The results of a study by Royakkers et al. in 151 heterogeneous ICU patients stated that serum and urine cystatin C were poor predictors of AKI as well as the need for renal replacement therapy (36). In contrast to the aforementioned findings, the results of some other relevant studies showed the superiority of serum or urine cystatin C to creatinine in the early detection of renal dysfunction in critically ill patients (30-33, 37-39). A probable explanation of this controversy lies in the different methodology of studies as well as inclusion/exclusion criteria, methods, and AKI definition. For instance, Herget-Rosenthal et al. included just patients with several risk factors for developing AKI (e.g. age > 70 years, cardiogenic or hemorrhagic shock, decompensated liver cirrhosis, diabetes, and sepsis) in their study and excluded patients admitted to ICU with AKI (37). In contrast to three studies that used the risk, injury, failure, loss, and end-stage (RIFLE) criteria for AKI identification (34, 36, 37), the detection of AKI in four studies was based on a single GFR cut-off of 80 mL/min/1.73 m^2 ^(30-33). Mazul-Sunko et al. defined AKI in their study as plasma creatinine > 267 μmol/L or urine output < 30 mL/h (35). In two subanalyses of EARLYARF trial by Nejat et al., AKI was defined by the acute kidney injury network (AKIN) criterion: an increase in plasma creatinine above baseline of at least 0.3 mg/dL (26.4 μmol/L) or 50% (38, 39). Cystatin C was measured by the immunonephelometric assay in all relevant studies (30-39) except our work in which determination of serum cystatin C was performed by the ELISA method. Interestingly, Dharnidharka et al. meta-analysis revealed that immunonephelometric methods of cystatin C measurement produced significantly greater correlations than other methods such as immunoturbidimetry or ELISA (R = 0.846 vs. 0.784, P < 0.001) (29). A jury of the international consensus conference in intensive care medicine held in 2007 concluded that although cystatin C is a promising marker of renal function in circumstances where alterations in the amount of creatinine tubular secretion may occur and where it is important to detect rapid changes in GFR, performing further clinical evaluations is necessary (40).

Unlike Ccr, the accuracy of serum creatinine for detection of GFR < 80 mL/min/1.73 m^2^ determined by the original and simplified MDRD formulas was statistically higher than that of serum cystatin C in our study. The MDRD equations were evaluated and validated primarily in non-ICU patients with chronic kidney disease and the data regarding the performance of MDRD formulas in ICU patients are limited. The results of Hoste et al. study questioned the applicability of MDRD formulas for the assessment of renal function in critically ill patients with normal serum creatinine (5). Comparing MDRD, modified Jelliffe, Mayo-Clinic and CG equations with the measured Ccr in 307 ICU adult patients indicated that modified Jelliffe had higher agreement with Ccr than other studied equations like MDRD (41). Two studies compared MDRD with a cystatin C-based formula in critically ill patients. A profound difference was found between the two GFR estimates. However, due to the absence of a gold standard method (an exogenous substance such as inulin or iothalamate) for GFR measurement in these two studies, it was not feasible to determine which formula was more appropriate for ICU patients (42, 43).

The performance of cystatin C as a marker of renal function in critically ill patients has been questioned by Wulkan et al. (44). A number of studies stated that overt as well as subclinical thyroid dysfunctions could significantly alter serum cystatin C level (13,14,45). On the other hand, nonthyroidal illness, a disorder typically manifests with low free triiodothyronine (T3) and normal or decreased thyroid-stimulating hormone (TSH) values, is very common in patients with critical illness. Thus, Wulkan et al. claimed that cystatin C was not a suitable marker of renal function in critically ill patients (44). However, Herget-Rosenthal et al. showed that neither low T3 nor T3/T4 syndromes markedly affected serum cystatin C in ICU patients (37). Therefore, it seems that nonthyroidal illness cannot be considered as a confounding factor of cystatin C level. However, this issue was not assessed in the current study.

The current study has several limitations. First, the study was performed in three ICUs of a single center; hence, the results might be vulnerable to a center effect and may not be reproduced in other settings. Second, the study lacked using an exogenous substance such as inulin or radioisotope as a real gold standard for more accurate estimation of GFR. Third, the exclusion criteria such as hemodynamic instability or hospital and ICU stay for more than one week confined the study to less seriously ill patients. This issue is supported by the low mean ± SD APACHE II score of the patients (8.14 ± 5.24). Finally, the detection of AKI was based on a single GFR cut-off of 80 mL/min/1.73 m^2^ rather than the pattern of serum creatinine or GFR alterations during several days of ICU stay (such as RIFLE criteria).

In conclusion, we demonstrated that serum cystatin C was not superior to serum creatinine in the early detection of renal dysfunction in critically ill patients. Considering the current controversies and lack of adequate data, further multicenter studies in large populations with sequential measurement of serum creatinine, cystatin C, and Ccr are warranted to elucidate the value of serum cystatin C as a marker of renal function in ICU patients.
